# Biosynthesis of storage compounds by *Rhodococcus jostii *RHA1 and global identification of genes involved in their metabolism

**DOI:** 10.1186/1471-2164-9-600

**Published:** 2008-12-12

**Authors:** Martín A Hernández, William W Mohn, Eliana Martínez, Enrique Rost, Adrián F Alvarez, Héctor M Alvarez

**Affiliations:** 1 Centro Regional de Investigación y Desarrollo Científico Tecnológico (CRIDECIT), Facultad de Ciencias Naturales, Universidad Nacional de la Patagonia San Juan Bosco, Km 4-Ciudad Universitaria, 9000 Comodoro Rivadavia, Chubut, Argentina; 2 Department of Microbiology and Immunology, Life Science Institute, University of British Columbia, Vancouver, BC, V6T 1Z3, Canada; 3 Facultad de Ingeniería, Universidad Nacional de la Patagonia San Juan Bosco, Km 4-Ciudad Universitaria, 9000 Comodoro Rivadavia, Chubut, Argentina; 4 Departamento de Genética Molecular, Instituto de Fisiología Celular, Universidad Nacional Autónoma de México, 04510 México D.F, México

## Abstract

**Background:**

Members of the genus *Rhodococcus *are frequently found in soil and other natural environments and are highly resistant to stresses common in those environments. The accumulation of storage compounds permits cells to survive and metabolically adapt during fluctuating environmental conditions. The purpose of this study was to perform a genome-wide bioinformatic analysis of key genes encoding metabolism of diverse storage compounds by *Rhodococcus jostii *RHA1 and to examine its ability to synthesize and accumulate triacylglycerols (TAG), wax esters, polyhydroxyalkanoates (PHA), glycogen and polyphosphate (PolyP).

**Results:**

We identified in the RHA1 genome: 14 genes encoding putative wax ester synthase/acyl-CoA:diacylglycerol acyltransferase enzymes (WS/DGATs) likely involved in TAG and wax esters biosynthesis; a total of 54 genes coding for putative lipase/esterase enzymes possibly involved in TAG and wax ester degradation; 3 sets of genes encoding PHA synthases and PHA depolymerases; 6 genes encoding key enzymes for glycogen metabolism, one gene coding for a putative polyphosphate kinase and 3 putative exopolyphosphatase genes. Where possible, key amino acid residues in the above proteins (generally in active sites, effectors binding sites or substrate binding sites) were identified in order to support gene identification. RHA1 cells grown under N-limiting conditions, accumulated TAG as the main storage compounds plus wax esters, PHA (with 3-hydroxybutyrate and 3-hydroxyvalerate monomers), glycogen and PolyP. *Rhodococcus *members were previously known to accumulate TAG, wax esters, PHAs and polyP, but this is the first report of glycogen accumulation in this genus.

**Conclusion:**

RHA1 possess key genes to accumulate diverse storage compounds. Under nitrogen-limiting conditions lipids are the principal storage compounds. An extensive capacity to synthesize and metabolize storage compounds appears to contribute versatility to RHA1 in its responses to environmental stresses.

## Background

Members of the genus *Rhodococcus *are widely distributed in natural environments, such as soil, water and marine sediments [1]. They belong to the non-sporulating and mycolic acid-rich group within the actinomycetes, together with other related genera, including *Mycobacterium*, *Nocardia*, *Corynebacterium *and *Gordonia*. The frequent occurrence of *Rhodococcus *sp. in arid sites like deserts around the world may reflect their adaptation to environments with extreme conditions. These microorganisms developed metabolic strategies to cope with such environments where nutrient-limitation is common. One of these mechanisms may be the accumulation of storage compounds that can be utilized by cells as endogenous carbon sources and electron donors during periods of nutritional scarcity.

*Rhodococcus jostii *RHA1 is a soil bacterium with the ability to degrade and transform polychlorinated biphenyls and other aromatic compounds [2]. The complete genome of strain RHA1 is available for screening and identification of genes and metabolic pathways. For this reason, *R. jostii *RHA1 is a good model organism for understanding the genetics and physiology of storage compound metabolism. Strain RHA1 possesses one of the largest bacterial genomes sequenced to date, containing 9.7 Mbp arranged in a linear chromosome (7,802,028 bp) and three linear plasmids: pRHL1 (1,123,075 bp), pRHL2 (442,536 bp) and pRHL3 (332,361 bp) [3].

The accumulation of storage lipids by actinomycetes, like members of *Mycobacterium*, *Rhodococcus*, *Nocardia *and *Streptomyces *is a well-established feature [4]. Members of these genera produce variable amounts of triacylglycerols (TAG) during growth on different carbon sources, and some species are able to accumulate very high levels of TAG in their cells [4,5]. This is the case for *Rhodococcus opacus *PD630, which accumulates TAG comprising up to 76% of its cellular dry weight after growth on gluconate [6]. The key enzymes involved in TAG and wax ester biosynthesis by bacteria are the wax ester synthase/acyl-CoA:diacylglycerol acyltransferase (WS/DGATs) enzymes. Kalscheuer and Steinbüchel identified a bifunctional enzyme from *Acinetobacter baylyi *sp. ADP1 that exhibits simultaneously both acyl-CoA:diacylglycerol acyltransferase and acyl-CoA:fatty alcohol acyltransferase (wax ester synthase) activities [7]. WS/DGATs catalyze the final step of TAG or wax ester biosynthesis in prokaryotes, using fatty acid CoA thioesters as substrates for esterification of diacylglycerols or long-chain fatty alcohols with the concomitant release of CoA [8]. Daniel et al. identified 15 putative WS/DGAT genes in *M. tuberculosis *strain H37Rv, which showed acyltransferase activity when expressed in *E. coli *[9]. In addition, 10 putative WS/DGAT genes were identified in *R. opacus *PD630, a species closely related to strain RHA1 [10]. A highly conserved motif HHxxxDG, which may be the catalytic site responsible for ester bond formation, is found in WS/DGATs from all known TAG-accumulating bacteria [4,8]. Stored bacterial TAG may be mobilized by cytoplasmic lipase/esterase enzymes, which may produce free acyl-residues available for generating energy through oxidation or as precursors for biosynthesis of other compounds. The lipases involved in endogenous TAG degradation in bacteria have been poorly studied. Deb et al. described a lipase enzyme which is responsible for the utilization of stored TAG during dormancy and reactivation of *M. tuberculosis *[11].

In addition to TAG, members of *Rhodococcus *are able to accumulate variable amounts of short chain length polyhydroxyalkanoates (PHA) during cultivation on different carbon sources [12-14]. Some *Rhodococcus *members accumulate a copolyester; poly(3-hydroxybutyrate-*co*-3-hydroxyvalerate) [poly(3HB-*co*-3HV)], and others a homopolyester of 3-hydroxybutyrate monomer units, from unrelated substrates such as gluconate, glucose and acetate [5,13]. PHA synthases represent the key enzymes of PHA biosynthesis, which catalyze the stereo-selective conversion of (*R*)-3-hydroxyacyl-CoA substrates to PHA with the concomitant release of CoA [15,16]. The *phaC*_*Rr *_gene from *R. ruber *is the only gene encoding a PHA synthase identified and cloned from a member of *Rhodococcus *[17]. The PhaC_*Rr *_enzyme is a short chain length class I PHA synthase, a class of enzymes comprised of only one type of subunit that utilize CoA thioesters of 3-hydroxy fatty acids with 3 to 5 carbon atoms [15]. Down-stream of the *R. ruber phaC*_*Rr *_gene is a gene (ORF4) coding for a putative PHA depolymerase, which is the key enzyme for PHA mobilization [18].

Other storage compounds produced by bacteria, besides lipids, are polyphosphates and carbohydrates like trehalose or glycogen. The latter is a glucose polymer with α 1,4 and α 1,6 linkages, usually considered a stored energy and carbon source [19]. Glycogen accumulation by bacteria often occurs during stationary phase [20]; although some bacterial species synthesize glycogen mainly during exponential growth phase [21]. In addition, glycogen may be accumulated by bacteria under different conditions, such as N-limited growth [20] or hyperosmotic stress [22]. The genetic aspects of glycogen biosynthesis and degradation have been studied intensively, mainly in *E. coli *[23]. In addition, there are studies on the genetics and biochemistry of glycogen metabolism in some actinomycetes, like *Mycobacterium *[21] and *Corynebacterium *[22,24]. So far as we know, there are no previous reports on glycogen biosynthesis by members of *Rhodococcus*. Key genes encoding enzymes involved in glycogen metabolism in several bacteria include (1) *glgC*, encoding an ADP-glucose pyrophosphorylase; (2) *glgB*, encoding a branching enzyme that may introduce α (1–6) linkages during glycogen synthesis [25]; (3) *glgX*, encoding a glycogen debranching enzyme; (4) *glgP*, encoding a protein that belongs to a structurally related and ubiquitous group of glucan-degrading enzymes, which catalyze the production of glucose-1-phosphate by the reversible cleavage of a-1,4 bonds at the non-reducing ends of polyglucans, such as maltodextrins, starch, and glycogen [26]; (5) *glgA*, encoding a glycogen synthase or glycosyltransferase; and (6) *glgE*, encoding an alpha amylase which is involved in glycogen degradation [21].

Polyphosphates (polyP) are additional storage compounds, which may help bacterial cells to respond and adapt to environmental stresses. This polymer is a linear chain of phosphate residues linked by phosphoanhydride bonds and synthesized in bacteria from ATP by polyphosphate kinases (PPKs), which are the key enzymes for polyP accumulation [27]. Some PPKs are also able to generate ATP from polyP and ADP (the reverse reaction). In contrast, other PPKs are only involved in the synthesis of PolyP, making exopolyphosphatases (PPXs) necessary to catalyze PolyP degradation [28]. PolyP may have multiple functions in bacterial cells, potentially enhancing their capacity to respond to oxidative stress, heat shock, osmotic stress, desiccation, and low-phosphate environments [29]. PolyP may also serve as an energy source to replace ATP and may be involved in the regulation of enzyme activities. The accumulation of polyP has been previously reported for *R. opacus *PD630 [6], which is a microorganism taxonomically related to *R. jostii *RHA1.

The purpose of this study was to examine the *R. jostii *RHA1 genome for the presence of key genes involved in storage compounds metabolism, like PHA, TAG, wax esters, glycogen and PolyP, and to analyze the physiological capability of strain RHA1 to accumulate these reserve substances.

## Methods

### Bacterial strain and growth conditions

*R. jostii *strain RHA1 was cultivated aerobically at 28°C in nutrient broth medium (NB) or in mineral salts medium (MSM) according to Schlegel et al. [30]. Sodium gluconate (1% w/v) or other substrates were used as sole carbon source. When N-limiting conditions were specified, the concentration of ammonium chloride in the MSM was reduced to 0.1 g/l (MSM0.1) to allow lipid accumulation [68]. Cells were harvested during exponential and stationary growth phases, washed with NaCl solution (0.85%, w/v) and lyophilised for chemical analyses.

### Extraction and analysis of lipids

Freeze-dried cells were extracted with methanol-chloroform (MeOH-CHCl_3_, 1:2, v/v). An aliquot of the whole cells extract was analyzed by thin layer chromatography (TLC) on 60F254 silica gel plates (Merck) applying *n-*hexane-diethyl ether-acetic acid (80:20:1, v/v/v) as a solvent system. Lipid fractions were revealed using iodine vapor. Tripalmitin and cetylpalmitate (Merck) were used as standards.

### Analysis of fatty acids and PHA

For qualitative and quantitative determination of fatty acids and PHA, 5–8 mg of lyophilised cells were subjected to methanolysis in the presence of 15% (v/v) sulphuric acid, and the acyl- and 3-hydroxyacyl-methylesters were analyzed by gas chromatography (GC) with an HP 5890 A gas chromatograph equipped with a Winnowed capillary column (30 m × 0.53 mm × 1 μm) and a flame ionization detector. The injection volume was 0.2 μl. Helium (13 mm/min) was used as carrier gas. The temperature of the injector and detector was 270°C and 320°C respectively. A temperature program was used for efficient separation of the methyl esters (90°C for 5 min, temperature increase of 6°C/min, 240°C for 17 min). For quantitative analysis, tridecanoic acid was used as internal standard.

### Extraction and analysis of cellular polysaccharide

The polysaccharide was isolated from freeze-dried cells by the classical alkali treatment described previously in several works [31-33] and visualized by two different TLC methods [22,24,34]. Total polysaccharide was determined by the phenol-sulfuric acid method [35]. Isolated cellular polysaccharide was boiled in 500 μl of 2 M trifluoroacetic acid (TFA) (90 min. 121°C), and the resulting components were analyzed by paper chromatography [36] and TLC methods as described above.

### Enzyme digestion of cellular polysaccharide

Since the isolated polysaccharide may be contaminated with other materials, a direct weight was not an accurate measurement of glycogen. Thus, samples (1 mg) were digested with α-amylase (10 UI) and amyloglucosidase (20 UI) in 50 mM sodium acetate buffer (pH 5) at 55°C for 2 hours. The amount of glucose under these conditions was taken as a measure of glycogen in cells. Glucose was determined by a specific glucose oxidase method [37].

### PolyP-staining method

For staining polyphosphate inclusions, a modification [38] of Methylene-blue staining (Loeffler) was used. Smears of RHA1 cells, cultivated in MSM0.1 with 1% gluconate, were fixed with gentle heat on glass microscopic slides and exposed to the following solutions followed in each case with light washing in distilled water: (1) Loeffler's methylene-blue solution, for 10 minutes; (2) sulfuric acid 1%, for 5 seconds; (3) Lugol's iodine solution, for 15 seconds; (4) aqueous safranine, for 2 minutes.

For the screening of the different storage compound metabolism genes, we used the available RHA1 genome database [39].

Database searches and alignments were carried out using BLAST 2.2.17 [40], CLUSTALW [41] and by comparison with other studies. Reference protein sequences were retrieved from the NCBI database. Identities were determined for alignments of full-length sequences.

## Results

### Key genes for PHA metabolism

We searched the RHA1 genome for genes involved in PHA metabolism. Such genes are often clustered in bacterial genomes [15,42]. We identified three chromosomal loci with genes involved in PHA metabolism, each containing both PHA synthase and PHA depolymerase genes (Table 1). These genes were not clustered with others encoding β-ketothiolase and NADPH-dependent acetoacetyl-CoA reductase. In this respect, RHA1 is like *R. ruber *and is unlike gram-negative short chain length PHA-accumulating bacteria [17]. It is unclear whether the small coding sequences located near the PHA synthase and depolymerase genes of RHA1 (ro00750, ro00752, ro03777 and ro04490) encode phasins, the structural proteins associated with PHA bodies. Neither these, nor any other RHA1 predicted proteins had high similarity to known phasins.

**Table 1:** Key genes encoding synthesis of TAG, wax esters, PHA, glycogen and PolyP by *R. jostii *RHA1.

*Gene ID*	*Gene name*	*Enzyme name*	*Length (aa)*	Accession number*	*Selected ortholog*	*Aminoacid identity (%)*
ro00753	*phaC*1	PHA synthase	568	YP_700746	PhaC_Rr_*R. ruber*	37
ro00754	*phaZ*1	PHA depolymerase	323	YP_700747	ORF4 *R. ruber*	12
ro03778	*phaC*2	PHA synthase	565	YP_703736	PhaC_Rr_*R. ruber*	39
ro03776	*phaZ*2	PHA depolymerase	271	YP_703734	ORF4 *R. ruber*	14
ro04491	*phaC*3	PHA synthase	565	YP_704435	PhaC_Rr_*R. ruber*	39
ro04489	*phaZ*3	PHA depolymerase	274	YP_704433	ORF4 *R. ruber*	14
ro05974	*glgA*	Probable glycosyltransferase	406	YP_705909	GlgA *Mycobacterium *sp. MCS	73
ro05975	*glgC*	ADP glucose Pyrophosphorylase	404	YP_705910	GlgC *M. tuberculosis *H37Rv	83
ro01447	*glgP*	Glycogen phosphorylase	862	YP_701423	GlgP *M. tuberculosis *H37Rv	69
ro01448	*glgE*	Probable alpha amylase	672	YP_701424	GlgE *M. smegmatis*	66
ro01449	*glgB*	Glycogen branching enzyme	732	YP_701425	GlgB *Mycobacterium *sp. MCS	70
ro01056	*glgX*	Probable glycogen debranching enzyme	753	YP_701041	GlgX *Mycobacterium *sp. MCS	75
ro00023	*atf*1	WS/DGAT	436	YP_700017	Atf1 *A. baylyi *ADP1	21
ro00024	*atf*2	WS/DGAT	477	YP_700018	Atf1 *A. baylyi *ADP1	24
ro00039	*atf*3	WS/DGAT	473	YP_700033	Atf1 *A. baylyi *ADP1	23
ro00087	*atf*4	WS/DGAT	461	YP_700081	Atf1 *A. baylyi *ADP1	32
ro00583	*atf*5	WS/DGAT	430	YP_700576	Atf1 *A. baylyi *ADP1	26
ro01601	*atf*6	WS/DGAT	453	YP_701572	Atf1 *A. baylyi *ADP1	38
ro02966	*atf*7	WS/DGAT	467	YP_702929	Atf1 *A. baylyi *ADP1	37
ro05356	*atf*8	WS/DGAT	463	YP_705294	Atf1 *A. baylyi *ADP1	37
ro05649	*atf*9	WS/DGAT	484	YP_705586	Atf1 *A. baylyi *ADP1	21
ro06332	*atf*10	WS/DGAT	474	YP_706267	Atf1 *A. baylyi *ADP1	27
ro06855	*atf*11	WS/DGAT	464	YP_706785	Atf1 *A. baylyi *ADP1	29
ro08369	*atf*12	WS/DGAT	301	YP_707571	Atf1 *A. baylyi *ADP1	35
ro08645	*atf*13	WS/DGAT	473	YP_707847	Atf1 *A. baylyi *ADP1	36
ro08660	*atf*14	WS/DGAT	497	YP_707862	Atf1 *A. baylyi *ADP1	24
ro06503	*ppk*	Polyphosphate kinase	734	YP_706434	Ppk *Pseudomonas aeruginosa*	39
ro02065	*ppx1*	Exopolyphosphatase	317	YP_702030	Ppx *Pseudomonas aeruginosa*	17
ro05780	*ppx2*	Exopolyphosphatase	314	YP_705716	Ppx *Pseudomonas aeruginosa*	17
ro06647	*ppx3*	Exopolyphosphatase	333	YP_706578	Ppx *Pseudomonas aeruginosa*	11

* NCRI protein data base

**Figure 1:**
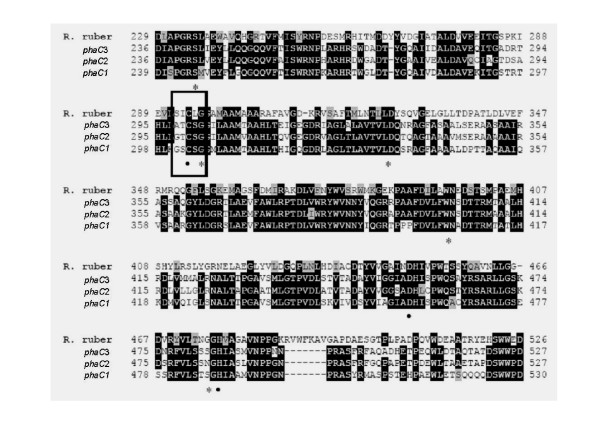
**Alignment of PHA-synthase genes of *R. jostii *RHA1 and *R. ruber***. Amino acid residues that are conserved in all the known PHA synthases are indicated below the sequences (*). Conserved residues probably involved in catalysis are shown (●). Putative lipase box is indicated with a rectangle.

The three PHA synthases encoded in the genome of RHA1 have 37% to 39% sequence identity to *R. ruber *PHA synthase (Table 1). Multiple alignments of the primary structures of 59 PHA synthases from 45 different bacteria, including *R. ruber*, showed the presence of eight highly conserved amino acid residues, which are important for the enzyme function [15]. These eight highly conserved amino acid residues are also present in the three PHA synthases of RHA1 (Fig. 1). All PHA synthases additionally contain a putative lipase box, G-X-(S/C)-X-G, in which the essential active-site serine of the lipases is replaced with a cysteine in the PHA-synthases [43,44]. The PHA synthases encoded by *phaC1 *and *phaC2 *in RHA1 contain the lipase box G-X-C-X-G, while that encoded by *phaC3 *has a modified lipase box, where the first glycine of the motif is replaced by alanine. Three amino acid residues are proposed to be required for catalytic activity of PHA synthase, presumably forming a catalytic triad, which is found in enzymes belonging to the superfamily of α/β-hydrolases [15,43]. The conserved residues C-294, D-449 and H-477 of *R. ruber *PHA synthase were proposed to be involved in covalent catalysis during PHA biosynthesis [15]. The three PHA synthases of strain RHA1 all contain the same catalytic triad.

The three putative PHA depolymerase proteins of RHA1 (Table 1) have high identities (45% to 46%) to *P. putida *KT2440 PHA depolymerase, whose function and characteristics have been studied previously [45]. Interestingly, the RHA1 enzymes have relatively low identity (12% to 14%) to *R. ruber *PHA depolymerase. Two of the RHA1 genes (*phaZ*2 and *phaZ*3) are predicted to encode intracellular PHA depolymerases, whereas *phaZ*1 seems to encode an extracellular PHA-depolymerase. This protein is longer than the other two proteins, with approximately 50 additional amino acid residues, which presumably correspond to a signal peptide necessary for secretion of the protein across the cytoplasmic membrane prior to its removal by signal peptidases [46]. Such a secreted PHA-depolymerase likely functions in catabolism of exogenous PHAs.

The three PHA depolymerases of strain RHA1 contain the lipase-box pentapeptide G-X-S-X-G in their sequences and the proposed catalytic triad (corresponding to S_102_, A_221_, H_248 _in the *P. putida *ortholog), with the serine residue within the lipase-box motif. The respective lipase boxes of the RHA1 PHA depolymerases (PhaZ1, GYSWG; PhaZ2, GLSWG; PhaZ3, GLSWG) aligned well with those of the PHA depolymerases of *R. ruber *(encoded by ORF4, GGSQG) and *P. putida *KT2440 (Pp5004, GVSWG). Thus, *R. jostii *RHA1 is equipped with the necessary genes/proteins for the biosynthesis, accumulation and mobilization of PHA.

### Key genes for TAG metabolism

**Figure 2:**
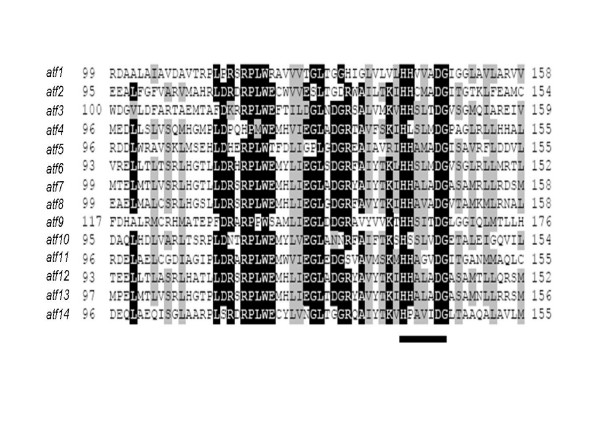
**Alignment of WS/DGAT genes of *R. jostii *RHA1**. The putative active site motif of WS/DGATs is shown with a bar below the sequences.

The key enzymes for the biosynthesis and mobilization of TAG in bacteria are WS/DGATs and lipase/esterases, respectively [4,8]. We used WS/DGAT genes from *A. baylyi *ADP1 [7], and *M. tuberculosis *[9] to screen the RHA1 genome for related genes. We identified 14 *atf *genes in the genome of RHA1 (Table 1). Some or all of these genes are likely involved in TAG biosynthesis in RHA1. All predicted RHA1 WS/DGATs have a length ranging from 430 to 497 amino acid residues, except *atf12 *product, which possesses 301 amino acid residues. Eleven of these genes contain the putative active site motif of WS/DGATs (HHxxxDG) (Fig. 2), while in *atf*4, *atf*10 and *atf*14, the second histidine of the motif is replaced by lysine, serine and proline; respectively. Eleven *atf *genes are located in the RHA1 chromosome, whereas *atf12, atf13 *and *atf14 *are located on plasmid pRHL1. The WS/DGAT genes of strain RHA1 are not located in operons with other genes involved in TAG metabolism, and they are widely distributed throughout the genome, which seems to be common in TAG-accumulating actinomycetes [8,9]. However, some of the 14 RHA1 WS/DGAT genes are adjacent or proximal to other genes likely involved in TAG or lipid metabolism. The *atf*6 gene is located up-stream of a probable esterase gene and the *atf*9 gene is located down-stream of a gene coding for a putative glycerol-3-phosphate acyltransferase, which is involved in the biosynthetic pathway of phospholipids and TAG. The *atf*11 gene is located down-stream of a gene encoding a lipase/esterase enzyme. The *atf*10 gene is located proximal to fatty acid desaturase genes. The predicted WS/DGAT proteins found in the genome database of RHA1 are 21% to 38% identical to Atf1 of *A. baylyi *ADP1.

The RHA1 genome encodes a broad repertoire for lipid degradation. We identified a total of 54 genes coding for putative lipase/esterase proteins (34 lipases and 20 esterases) in the genome (Table 2). These genes likely involved in neutral lipid degradation are widely distributed throughout the genome. Twelve of these genes are located on plasmids, nine on pRHL1 and three on pRHL2, while the rest of the putative lipase/esterase genes are located in the RHA1 chromosome. This is the first report of the occurrence of WS/DGAT genes and lipase/esterase genes on plasmids. Overall, analysis of the RHA1 genome indicated that this bacterium is endowed with broad capacity for TAG biosynthesis and degradation, with potentially redundant genes and enzymes.

**Table 2:** Lipases and esterases enzymes from RHA1 and their cellular localization.

Lipases*	*Subcelular localization*	*Esterases	Subcelular localization
ro00140	Unknown	ro00968	cytoplasmic
ro00410	extracelular	ro01280	cytoplasmic
ro01215	Unknown	ro01602	cytoplasmic
ro01244	cytoplasmic	ro01804	cytoplasmic
ro01897	Unknown	ro03129	cytoplasmic
ro02361	cytoplasmic	ro03795	cytoplasmic
ro02486	cytoplasmic	ro03905	cytoplasmic
ro02663	Unknown	ro04327	cytoplasmic
ro03099	cytoplasmic	ro04513	Unknown
ro03436	cytoplasmic	ro04768	cytoplasmic
ro04027	Unknown	ro04911	cytoplasmic
ro04081	cytoplasmic	ro05142	cytoplasmic
ro04106	Unknown	ro06006	cytoplasmic
ro04422	Unknown	ro06374	cytoplasmic
ro04722	Unknown	ro06680	cytoplasmic
ro05038	Unknown	ro06687	cytoplasmic
ro05138	cytoplasmic	ro06780	cytoplasmic
ro05310	cytoplasmic	ro07106	cytoplasmic
ro05456	Unknown	ro08559	cytoplasmic
ro06108	Unknown	ro10311	cytoplasmic
ro06372	Unknown	-	-
ro06856	cytoplasmic	-	-
ro06995	Unknown	-	-
ro07162	Unknown	-	-
ro08131	Unknown	-	-
ro08132	extracelular	-	-
ro08421	Unknown	-	-
ro08422	Unknown	-	-
ro09010	cytoplasmic	-	-
ro09026	cytoplasmic	-	-
ro09036	cytoplasmic	-	-
ro09094	Unknown	-	-
ro10223	cytoplasmic	-	-
ro10264	cytoplasmic	-	-

* Gene ID

### Accumulation of storage lipids

**Figure 3:**
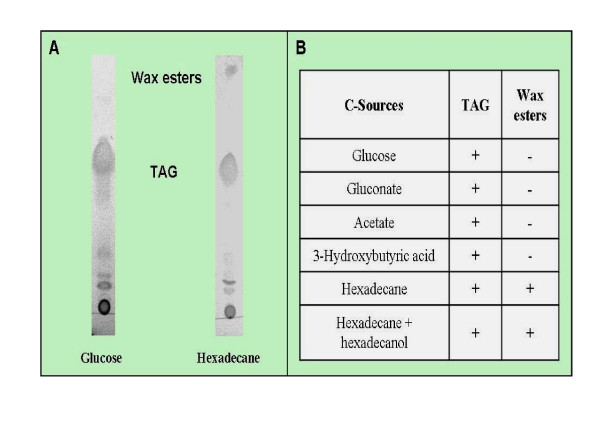
**Accumulation of TAG and wax esters by *R. jostii *RHA1 as revealed by TLC analyses**. A, TLC of lipids extracted from gluconate and hexadecane-grown cells; B, Occurrence of TAG and wax esters in cells after growth on different carbon sources.

We investigated the accumulation of PHA, TAG and wax esters during cultivation of RHA1 in the presence of different carbon sources under nitrogen-limiting conditions. Nitrogen-limitation promotes the biosynthesis and accumulation of lipids by bacteria [5]. *R. jostii *RHA1 was able to synthesize and accumulate mainly TAG and minor amounts of a short-chain length copolyester, containing 3-hydroxybutyric acid (3HB) and 3-hydroxyvaleric acid (3HV) as monomer units (Fig. 3 and Table 3). When the cells were cultivated on hexadecane or on a mixture of hexadecane-hexadecanol, they produced wax esters in addition to TAG, as revealed by TLC (Fig. 3). The main fatty acids produced by hexadecane- and hexadecane-hexadecanol-grown cells were related to the chain length of those substrates, as well as to β-oxidation products of those substrates (Table 3). During cultivation on glucose, sodium gluconate, sodium acetate and 3-hydroxibutyric acid, which all have to be degraded to acetyl-CoA before they enter other metabolic pathways, RHA1 accumulated TAG, but no wax esters were detected by TLC analysis (Fig. 3). Hexadecanoic acid (C_16:0_) and octadecenoic acid (C_18:1_) were always the predominant fatty acids occurring in the accumulated lipids after cultivation on the above four carbon sources (Table 3). In addition, the proportion of fatty acids with odd numbers of carbon atoms was relatively high after cultivation on those four substrates, ranging from 11.1% to 31.0% of the total fatty acids. The copolyester accumulated by strain RHA1; (poly-3HB-*co*-3HV), contained higher relative amounts of the C_5 _monomer (3HV) than the C_4 _one (3HB), as revealed by GC analysis (Table 3). When cells were grown on acetate or 3-hydroxybutyric acid as sole carbon sources, they produced higher relative amounts of 3HB units in the copolyester in comparison with those cells cultivated on glucose or gluconate (Table 3).

**Table 3:** PHA and fatty acid content (%) of *R. jostii *RHA1 after cultivation in MSM0.1 with different carbon sources*.

	Glucose	Gluconate	Acetate	3HB	Hexadecane	Hexadecane + hexadecanol
PHA	2.2	7.6	3.5	5.8	tr	tr
3HB	20.0	15.3	32.9	27.3	nd	nd
3HV	80.0	84.7	67.1	72.7	tr	tr
Fatty acids	48.4	56.9	21.2	32.5	30.4	7.0
C_14:0_	1.3	1.3	1.6	1.5	5.6	6.3
C_15:0_	4.4	3.7	2.3	1.6	nd	nd
C_16:0_	31.0	35.5	34.6	38.4	62.6	76.5
C_16:1_	10.1	9.1	13.3	13.1	29.5	17.2
C_17:0_	11.8	11.0	4.9	4.0	nd	nd
C_17:1_	14.8	9.8	9.4	5.5	1.0	nd
C_18:0_	9.3	13.4	9.1	12.3	nd	nd
C_18:1_	17.3	16.2	24.8	23.6	1.3	nd

* Total PHA and Fatty acids are expressed as % of cellular dry weight. Relative proportions of individual PHAs and fatty acids are expressed as % (w/w). Abbreviations: nd, not detected; tr, traces.

### Key genes for glycogen metabolism

Six *glg *genes encoding key enzymes for glycogen metabolism were identified in RHA1 (Table 1). Three of these genes, *glgP*, *glgE *and *glgB*, are in a cluster, which is conserved in glycogen-accumulating *M. smegmatis *[21] and *M. tuberculosis*. Another two of the genes, *glgA *and *glgC*, are adjacent at a different locus and divergently oriented. The sixth gene, *glgX*, is at a third locus, adjacent to another carbohydrate metabolism gene, which encodes a 1–4 α D-glucan 1-α-D-glucosylmutase. Several other genes associated with carbohydrate metabolism occur in the RHA1 genome, encoding glycosyltransferases, glycosidases, sugar transporters, glucose dehydrogenases, sugar kinases, glycohydrolases and aldolases.

The *glgC *gene of RHA1 encodes a 404-aa ADP-glucose pyrophosphorylase. This protein is 43%, 73%, 83% and 91% identical to orthologs from *E. coli *APEC O1, *C. glutamicum*, *M. tuberculosis *and *N. farcinica *IFM10152, respectively. In the *E. coli *ADP-glucose pyrophosphorylase, Tyr-114 and Lys-195 residues were identified as important in ATP and glucose-1-phosphate binding, respectively [47], and Asp-142 and Arg-32 residues were proposed to be involved in catalysis [48,49]. These four residues are conserved in GlgC of RHA1 (Tyr-99, Lys-180, Asp-128 and Arg-19, respectively). In addition, we identified a sequence motif, RAKPAV (residues 27–32 in GlgC of RHA1), which is present in all ADP-glucose pyrophosphorylases and is considered important for activator binding [24,50,51]. This enzyme is considered essential for glycogen synthesis and its regulation [20].

The *glgB *gene of RHA1 encodes a branching enzyme that introduces α (1–6) linkages during glycogen synthesis. In only a few bacteria, has this glucan branching enzyme been studied in detail. Alignment of RHA1 GlgB with orthologs allowed identification of four conserved regions as well as the conserved amino acids typical for members of family 13 glycoside hydrolases (alpha amylase family), whose properties have been studied intensively. The RHA1 GlgB catalytic residues were identified as Asp-407, Glu-461 and Asp-529, corresponding to the amino acids Asp-308, Glu-351 and Asp-419 of the *Bacillus stearothermophilus *enzyme, previously shown to constitute the catalytic triad [25,52]. These residues are also present in the orthologous enzymes of *E. coli *and *Mycobacterium *sp. MCS, the latter 70% identical to that of RHA1. Moreover, four other conserved amino acids, Asp-338, His-343, Arg-406 and His-528, likely involved in substrate binding, were identified in RHA1 GlgB, based on the amino acid sequence alignment.

The *glgX *gene of RHA1 encodes a glycogen debranching enzyme. This 753-aa protein has a predicted molecular mass of 83 kDa. The protein has high identities with debranching enzymes from other actinomycetes, such as *Mycobacterium *sp. MCS (75%) and *C. glutamicum *ATCC 13032 (66%), as well as the *E. coli *debranching enzyme GlgX (43%), which has been functionally characterized [53].

**Figure 4:**
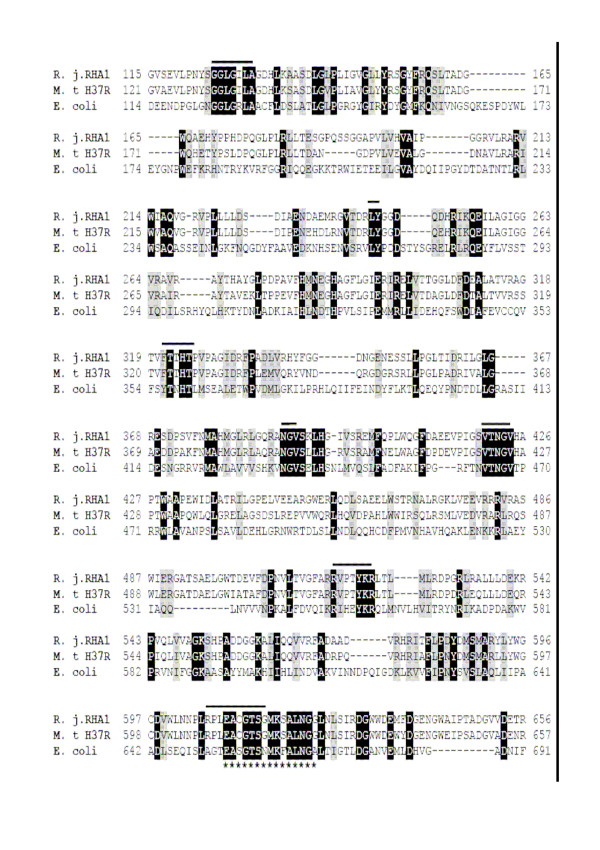
**Partial view of amino acid sequence alignment of glucan phosphorylases**. The identical amino acids in the three sequences are indicated by black background. The deduced active sites are indicated by lines, and the putative pyridoxal-phosphate cofactor binding site is indicated by asterisks.

The *glgP *gene of RHA1 encodes an 862-aa glycogen phosphorylase with high identity to orthologs from other microorganisms, such as *M. tuberculosis *H37Rv (69%). GlgP of RHA1 contains conserved features of other glycogen phosphorylases (Fig. 4), including proposed active sites and a signature sequence (EACGTSGMKSALNG) consistent with a pyridoxal-phosphate cofactor binding site [54].

The *glgA *gene of RHA1 encodes a 406-aa glycosyltransferase with a calculated molecular weight of 42.9 kDa. This protein has 73% identity to a glycogen synthase from *Mycobacterium *sp. MCS and 25% identity to one from *E. coli *APEC O1. We were not able to find the proposed active site described for the well-studied glycogen synthase of *E. coli *[55]. However, based on a previous multiple sequence alignment of selected glycosyltransferases [56], we identified an E-X_7-_E motif in GlgA of RHA1 that is shared among glycogen synthases of prokaryotes and eukaryotes (data not shown). This E-*X*_7_-E motif is part of the active site of eukaryotic glycogen synthases and both conserved glutamic acid residues (E) are involved in catalysis [56].

Finally, *glgE *of RHA1 encodes a 672-aa alpha amylase. The primary sequence of this protein has 66% identity and 77% similarity to that of a putative glucanase (GlgE) of *M. smegmatis*. An *M. smegmatis *temperature-sensitive *glgE *mutant showed an abnormal accumulation of glycogen during exponential growth, in comparison to the wild-type strain [21]. A sequence alignment (not shown) shows that the histidine residue that was mutated (His349) in *M. smegmatis *is conserved in GlgE of RHA1 (His322).

### Glycogen accumulation by RHA1

**Figure 5:**
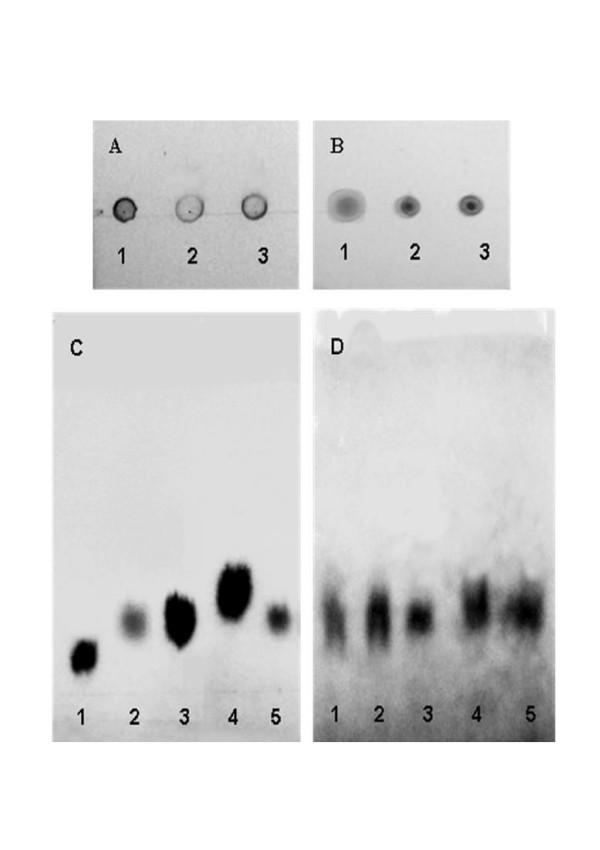
**Glycogen chromatographic analysis**. TLC analysis of glycogen in cells grown on Gluconate (A) and NB (B), with glycogen standard (1) and cellular glycogen in stationary (2) and exponential (3) growth phases. Analytical paper chromatography of RHA1 carbohydrates hydrolyzed with TFA (C), with maltose standard (1), hydrolyzed RHA1 sample (2), glucose standard (3), xylose standard (4) and hydrolyzed glycogen standard (5), or hydrolyzed with alpha amylase/amyloglucosidase (D), with stationary- and exponential-phase RHA1 grown on NB (1 and 2), glucose standard (3), stationary- and exponential-phase RHA1 grown on gluconate (4 and 5).

After cultivation of cells on both, nutrient broth and minimal salts medium with gluconate as sole carbon source, a phenol-sulphuric acid reactive material was detected in RHA1 using two different TLC methods with a commercial glycogen as standard (Fig. 5A and 5B). Samples of isolated polysaccharide from RHA1 digested with TFA were analyzed by paper chromatography and TLC methods. These analyses showed that glucose was the sole sugar component of the polysaccharide. Identical results were obtained with hydrolyzed glycogen standard (Fig. 5C). These results indicate that the phenol-sulphuric acid reactive material isolated from strain RHA1 is a glucose polymer.

The glucose polymer (glucan) was also characterized and quantified by enzymatic digestion and comparison to the commercial glycogen. Hydrolysis with only with α amylase, which catalyzes the endohydrolysis of α 1,4 D-glucosidic linkages, released glucose, maltose plus oligosaccharides that were identified by TLC and paper chromatography (data not shown). Hydrolysis with α amylase plus amyloglucosidase, which catalyzes hydrolysis of terminal α 1,4 D-glucose residues and α 1,6 D-glucosidic bonds, converted total carbohydrate to free glucose (Fig. 5D), indicating the presence of both α 1,4 and α 1,6 bonds. Taken together, these results indicate that the polysaccharide accumulated by RHA1 is glycogen.

Glycogen was detected in both media (NB and MSM0.1) during exponential and stationary growth phases (Table 4). In both media, glycogen accumulation during exponential growth phase was higher than during stationary phase, especially in MSM0.1 medium with 1% gluconate as the sole carbon source.

**Table 4:** Glycogen accumulation by *R. jostii *RHA1 grown on NB and MSM0.1 with gluconate during different growth phases.*

Media and growth phase	Glycogen (% CDW)
NB Exponential growth phase	2.3 ± 0.4
NB Stationary growth phase	1.9 ± 0.2
MSM0.1 + Gluconate 1% Exponential growth phase	3.5 ± 0.01
MSM0.1 + Gluconate 1% Stationary growth phase	2.05 ± 0.3

*All data were recorded as means of three separate assays ± standard deviation.

### Key genes for PolyP metabolism and accumulation of PolyP

A single *ppk *gene was identified in RHA1 (Table 1). This gene was adjacent to other functionally unrelated genes. The *ppk *gene product has three regions and two histidine residues (H485 and H504) (data not shown) that may be involved in the enzymatic activity and are conserved in orthologs from *P. aeruginosa *[57] and *E. coli *[58].

On the other hand, three *ppx *genes encoding putative cytoplasmic exopolyphosphatases (Ppx-related proteins) were found widely distributed in the RHA1 genome. Exopolyphosphatase enzymes are normally involved in PolyP degradation. Ppx enzymes from *E. coli *[59] and *P. aeruginosa *(28, 60) have been cloned, sequenced and well-characterized. The RHA1 Ppx proteins range from 11% to 31% identical to that of *P. aeruginosa *PPX (Table 1).

**Figure 6:**
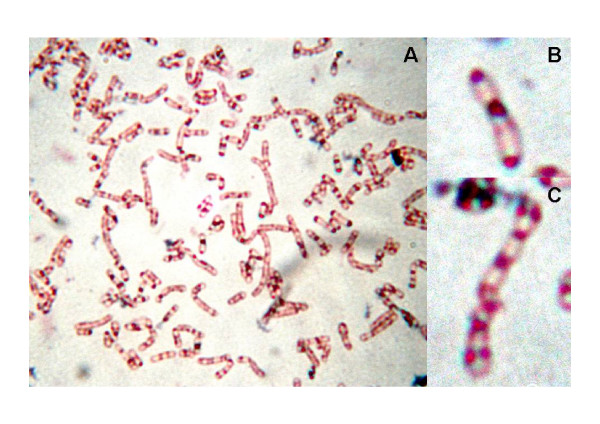
**Modified Loeffler's methylene blue staining of *Rhodococcus jostii *RHA1 grown on MSM0.1 with gluconate 1% (w/v)**. (A) General view of cells by optic microscopy (1,000 ×). (B) and (C) Magnified views of cells showing dark stained polyP inclusions and lightly stained cytoplasm. These photographs have been digitally processed to increase magnification (Original magnification: 1,000 ×).

The occurrence of putative a *ppk *gene in the genome of strain RHA1 suggests that this bacterium has the potential to synthesize PolyP, like other members of *Rhodococcus *[6]. In addition, PolyP is likely mobilized by RHA1 cells using PPX-related proteins. In order to confirm the ability of strain RHA1 to accumulate PolyP, we analyzed cells by microscopy after cultivation on gluconate as the sole carbon source under N-limiting conditions. PolyP-bodies were observed in RHA1 cells as dark granules in contrast to a light red cell background by means of a modification of the methylene-blue staining method (Fig. 6). Identical results were observed with cells of *R. opacus *PD630 used as positive control, which was previously shown to accumulate PolyP [6].

## Discussion

In this study, we report the ability of *R. jostii *RHA1 to synthesize and accumulate different storage compounds, including poly(3HB-*co*-3HV), TAG, wax esters, glycogen and polyP. We found that the RHA1 genome is remarkably rich in genes involved in storage lipid metabolism, with genes encoding 14 WS/DGATs, 54 lipases/esterases and three sets of PHA synthases/depolymerases. These results agree with previous observations in other actinomycetes, like *M. tuberculosis*, which devotes a large portion of its genome to genes involved in lipid metabolism [61].

Similarly to RHA1, *M. tuberculosis *contains 15 *atf *genes encoding WS/DGATs, [9]. Some of these genes, like *rv3130c*, show the highest induction and activity during hypoxia [9,62]. Multiple *atf *genes were also identified in the genomes of other actinomycetes, including three in *S. coelicolor *[63], 10 in *R. opacus *[10], 12 in *M. bovis *AF2122/97, 8 in *M. smegmatis *mc^2^155, 5 in *N. farcinica *IFM 10152 and 4 in *Nocardioides *sp. JS614 [8]. By contrast, TAG/wax esters accumulating gram-negative bacteria seem to have single *atf *genes, as is the case for *A. baylyi *sp. ADP1, *Psychrobacter *sp. and *Polaromonas *sp. [8]. In general, actinomycetes accumulate higher amounts of TAG than gram-negative bacteria, and they have a high lipid content in different cellular structures, such as cell envelope or lipid inclusions [4,13].

The multiplicity of *atf *genes in *Rhodococcus *and other actinomycetes, and their ability to synthesize TAGs as main storage lipids, suggest an important role of these lipids in the physiology of these organisms and their ability to cope with adverse environments. TAGs are excellent reserve materials for several reasons. Their extreme hydrophobicity allows their accumulation in large amounts in cells without changing the osmolarity of the cytoplasm. Oxidation of TAGs produces the relatively high yields of energy in comparison with other storage compounds such as PHA and carbohydrates, since the carbon atoms of TAG acyl residues are in a very reduced state [5]. Moreover, TAGs may play other important functions in cells of actinomycetes, including (1) regulating the fatty acid composition of lipid membranes, (2) as a sink for reducing equivalents in cells when the terminal acceptor is not sufficiently supplied under low oxygen conditions, (3) as source of precursors for biosynthesis and turnover of mycolic acids during adaptation to changing environmental conditions, (4) as a reservoir of metabolic water for cells under water stress conditions, (5) as an agent to detoxify free fatty acids or unusual fatty acids that may disturb membrane fluidity during catabolism of hydrocarbons, or (6) as a source of precursors for the biosynthesis of antibiotics [4,64-67].

The RHA1 genome contains three sets of genes involved in PHA metabolism. However, PHAs represent only minor components of RHA1 storage lipids under the conditions used in this study. This is a common feature of all *Rhodococcus *members so far investigated, except for *R. ruber*, which is able to accumulate considerable amounts of both PHA and TAG (approx. 1:1) [14,68]. PHA may also serve as an endogenous source of carbon and energy for cells, although strain RHA1 possesses higher amounts of TAG, which are energetically more efficient than PHA, as discussed above. Thus, the importance of PHA for this function is relative. On the other hand, PHA, with C more oxidized than in TAG, may help cells to balance their redox state in environments with fluctuating conditions like soil.

In addition to TAG, wax esters were detected in hexadecane- and hexadecane/hexadecanol-grown cells. It was evident that fatty acid plus alcohol intermediates were available in these cells for the biosynthesis of wax esters in addition to TAG by the WS/DGATs, which are normally bifunctional enzymes involved in the synthesis of both storage lipids. It is known that fatty alcohols occur as intermediates during the degradation of alkanes. In contrast, no wax esters but TAG were detected after growth of cells on glucose, gluconate, acetate and 3-hydroxybutyric acid. These results suggest that strain RHA1 is not capable of providing fatty alcohols as substrates during growth on these carbon sources, which have to be degraded to acetyl-CoA before they enter other metabolic pathways.

The high proportion of odd-numbered fatty acids and the 3HV monomer in the stored TAG and PHA, respectively, suggest that strain RHA1 possesses an efficient mechanism for production of the intermediate propionyl-CoA, which was presumably utilized as precursor for the biosynthesis of fatty acids containing an odd-number of carbon atoms and the 3HV units of the copolyester. In addition, the growth of cells on acetate or 3-hydroxybutyric acid as sole carbon sources induced higher relative amounts of 3HB units in the copolyester in comparison with those cells cultivated on glucose or gluconate. Acetate may favor the production of 3HB units, since this monomer is normally synthesized by condensation of two acetyl-CoA residues. On the other hand, 3-hydroxybutyric acid used as carbon source may be mainly degraded before entering the biosynthesis pathway for PHA, although a small part may be used directly as monomer unit for the copolyester, likely after activation as 3-hydroxybutyryl-CoA.

The results of this study indicate that *R. jostii *RHA1 also possess the ability to synthesize and accumulate glycogen as an additional storage compound. To our knowledge this is the first report on glycogen accumulation by a *Rhodococcus *member. However, glycogen accumulation has been reported to occur in other actinomycetes, such as members of *Mycobacterium *[21,31] and *Corynebacterium *[24]. The RHA1 genome contains all necessary structural genes for the biosynthesis and degradation of glycogen. In addition, in this study, we chemically characterized the glycogen accumulated by strain RHA1. In contrast to many bacterial species which accumulate glycogen only during stationary phase or limited growth conditions [20], RHA1 accumulated more glycogen during exponential growth phase. Glycogen accumulation during exponential growth phase has been observed in other actinomycetes, such as *M. smegmatis *[21,31] and *C. glutamicum *[24]. The glycogen content in strain RHA1 decreased during stationary phase relative to exponential growth phase, under the culture conditions used in this study. The total content of glycogen in cells of strain RHA1 was rather low in comparison with the amount of accumulated TAG. We suggest that the biosynthesis pathways of PHA, TAG and glycogen compete for common precursors, which are used by cells preferentially for TAG biosynthesis rather than for PHA and glycogen accumulation. However, the metabolic relationship in strain RHA1 between these storage compounds must be investigated in further detail. Moreover, more studies are necessary to determine the role of glycogen in the RHA1 physiology. The content of glycogen in cells may be the result of a well coordinated process of synthesis and degradation as occur in *M. smegmatis *in which, glycogen has been proposed as a carbon capacitor for glycolysis during exponential growth [21]. Glycogen may have a role as metabolic intermediate since it is accumulated mainly during the exponential growth phase by cells and is mobilized later in the stationary phase, or may act as part of a sensing/signalling mechanism. Interestingly, Persson et al. [69] proposed that the expression of some genes involved in the response of *E. coli *to carbon starvation or stationary phase, like that encoding the universal stress protein (*uspA*), is regulated by glycolytic intermediates such as fructose-6-phosphate. Alteration in the pool size of phosphorylated sugars of the upper glycolytic pathway may ensure expression of stress proteins preceding the complete depletion of the external carbon source and growth arrest [69]. Thus, glycogen formation may act to attenuate phosphorylated sugar signals and to protect cells from sudden increases in fluxes of sugars.

The occurrence of one gene encoding Ppk and three putative Ppxs in the RHA1 genome and the presence of abundant intracellular metachromatic granules indicated that strain RHA1 possesses the ability to accumulate polyP. The occurrence of polyP has been described in other actinomycetes [6], such as members of *Corynebacterium *and *Mycobacterium *after cultivation on nitrogen-limited [70] or phosphate-rich media [71]. The availability of this high-energy phosphate polymer may enhance the capacity of strain RHA1 to survive in soil environments by providing phosphate for biosynthesis, maintenance energy or an osmoprotectant.

## Conclusion

This study evaluates the global capacity for storage compound metabolism by RHA1 and demonstrates the functionality of the biosynthetic pathways. This knowledge constitutes a framework for additional studies to determine the physiological and ecological functions and significance of these storage compounds for RHA1 and related bacteria. The occurrence of diverse storage compounds in RHA1 emphasizes the complexity of the physiology and biochemistry of this heterotrophic soil bacterium. Strain RHA1 is endowed with diverse sets of genes and enzymes for the metabolism of storage compounds, which seem to be redundant for storage lipid metabolism and polyP synthesis but not for glycogen metabolism or polyP degradation. Individual isoforms of enzymes potentially have different substrate specificity, may play distinct functional roles in the pathways of glycerolipid biosynthesis or may be differentially expressed under various environmental conditions. Additional information about gene or protein expression and enzyme activities will be required to distinguish specific roles of isoenzymes in strain RHA1. This complexity reflects the richness, diversity and versatility of lipid metabolism in RHA1 and related lipid-rich bacteria. The ability of these bacteria to accumulate diverse storage compounds may permit cells to rapidly respond to stress and to maintain active its metabolism under fluctuating environmental conditions, providing them with an adaptive advantage over less versatile bacteria.

## Authors' contributions

MAH carried out the experimental studies, participated in the sequence analysis and alignment, helped in the design of the study and drafted the manuscript. EM and ER participated in the experimental studies on storage lipids. AFA participated in the acquisition of data, sequence analysis and alignment, and interpretation of data. WWM participated in coordination of the study, interpretation of data and helped to draft the manuscript. HMA conceived the study and participated in its design and coordination, interpretation of data, and helped to draft manuscript. All authors read and approved the final manuscript.
